# A System of Cytokines Encapsulated in ExtraCellular Vesicles

**DOI:** 10.1038/s41598-018-27190-x

**Published:** 2018-06-12

**Authors:** Wendy Fitzgerald, Michael L. Freeman, Michael M. Lederman, Elena Vasilieva, Roberto Romero, Leonid Margolis

**Affiliations:** 1 Section of Intercellular Interactions, Eunice Kennedy-Shriver National Institute of Child Health and Human Development, National Institutes of Health, Bethesda, USA; 2 Case Western Reserve University, Cleveland, USA; 3 Evdokimov Moscow University of Medicine and Dentistry, Moscow, Russia; 4 Neonatology Branch, Eunice Kennedy-Shriver National Institute of Child Health and Human Development, National Institutes of Health, Detroit, USA

## Abstract

Cytokines are soluble factors that mediate cell–cell communications in multicellular organisms. Recently, another system of cell–cell communication was discovered, which is mediated by extracellular vesicles (EVs). Here, we demonstrate that these two systems are not strictly separated, as many cytokines *in vitro*, *ex vivo*, and *in vivo* are released in EV-encapsulated forms and are capable of eliciting biological effects upon contact with sensitive cells. Association with EVs is not necessarily a property of a particular cytokine but rather of a biological system and can be changed upon system activation. EV-encapsulated cytokines were not detected by standard cytokine assays. Deciphering the regulatory mechanisms of EV-encapsulation will lead to a better understanding of cell–cell communications in health and disease.

## Introduction

Multicellular organisms are sustained by complex systems of intercellular communications. These include: cell-cell contacts, soluble factors (cytokines, hormones, neurotransmitters) and recently discovered extracellular vesicles (EVs) that carry proteins, lipids, and micro RNAs^1^. Cytokines are generally considered to function as classical soluble molecules. Here, however, we report on a system of bioactive cytokines that are released being encapsulated in extracellular vesicles.

We systematically analyzed the association between 33 cytokines and EVs in eight *in vitro*, *ex vivo* and *in vivo* biological systems (cultured T cells, cultured monocytes, explants of tonsillar, cervical, placental villous, and amnion tissues, amniotic fluid, and blood plasma of healthy volunteers). We found that a cytokine could be released predominantly either in soluble or in EV-associated form depending on the biological system. Moreover, upon stimulation, the pattern of encapsulation is changed depending on the stimulus, suggesting that the encapsulation of cytokines in EVs is not simply the property of a particular cytokine, but rather a tight biological process. Importantly, EVs carrying cytokines are biologically active upon interacting with sensitive cells. The system of EV-encapsulated cytokines, which is not revealed by traditional assays, may play a significant role in health and disease.

## Results

### Free (soluble) and EV-associated cytokines

Culture medium from all cultured cells and explants as well as body fluids contained different amounts of EVs with mean sizes ranging from ~120–220 nm (Supplementary Table 1). We separated EVs from culture medium or body fluids using ExoQuick (for blood plasma) or ExoQuick TC (for culture supernatants and other body fluids). Cytokines were evaluated in both EVs and soluble fractions using a Luminex platform. To evaluate cytokines encapsulated in EVs they were lysed with 1% Triton X and the resultant cytokine concentration (released both from the surface and from inside of EVs) was evaluated. The amounts of encapsulated cytokines were calculated by subtracting the amounts of surface-attached cytokines, measured before adding the detergent, from that detected after EVs were lysed. Trypsin treatment of EVs was used to verify cytokines were on the surface or internal to EVs. Trypsinization resulted in the decrease of surface-bound cytokines, but the encapsulated ones remain largely intact: measurement of cytokines following trypsin treatment of tonsil-released EVs removed all but 26.3 ± 9.1% of the surface-associated cytokines, while inside 95.1 ± 18.8% of the initial cytokine amount remained (n = 5). Likewise, after trypsin treatment of placental villi-released EVs 15.5 ± 11.4% of cytokines were left on the surface but 94.4 ± 27.4% remained encapsulated compared to untreated EVs (n = 2). To verify that free cytokines are not co-precipitated with EVs when using ExoQuick, we spiked fresh culture medium with recombinant cytokines of known quantities, isolated EVs with ExoQuick, and measured cytokines in the EV fraction. Only trace amounts of cytokines were found in EV fractions (on average 0.24% of total cytokine). Additionally, we verified that cytokine association with the surface of EVs was not the result of binding of free cytokines to EVs when we spiked EVs from Jurkat cells with recombinant cytokines and again performed ExoQuick isolation and cytokine measurements on supernatant and EV fractions. Again we detected virtually no EV-associated cytokines (on average 0.76% of total cytokine).

### Free vs. EV-associated cytokines in different biological systems

**Figure 1:**
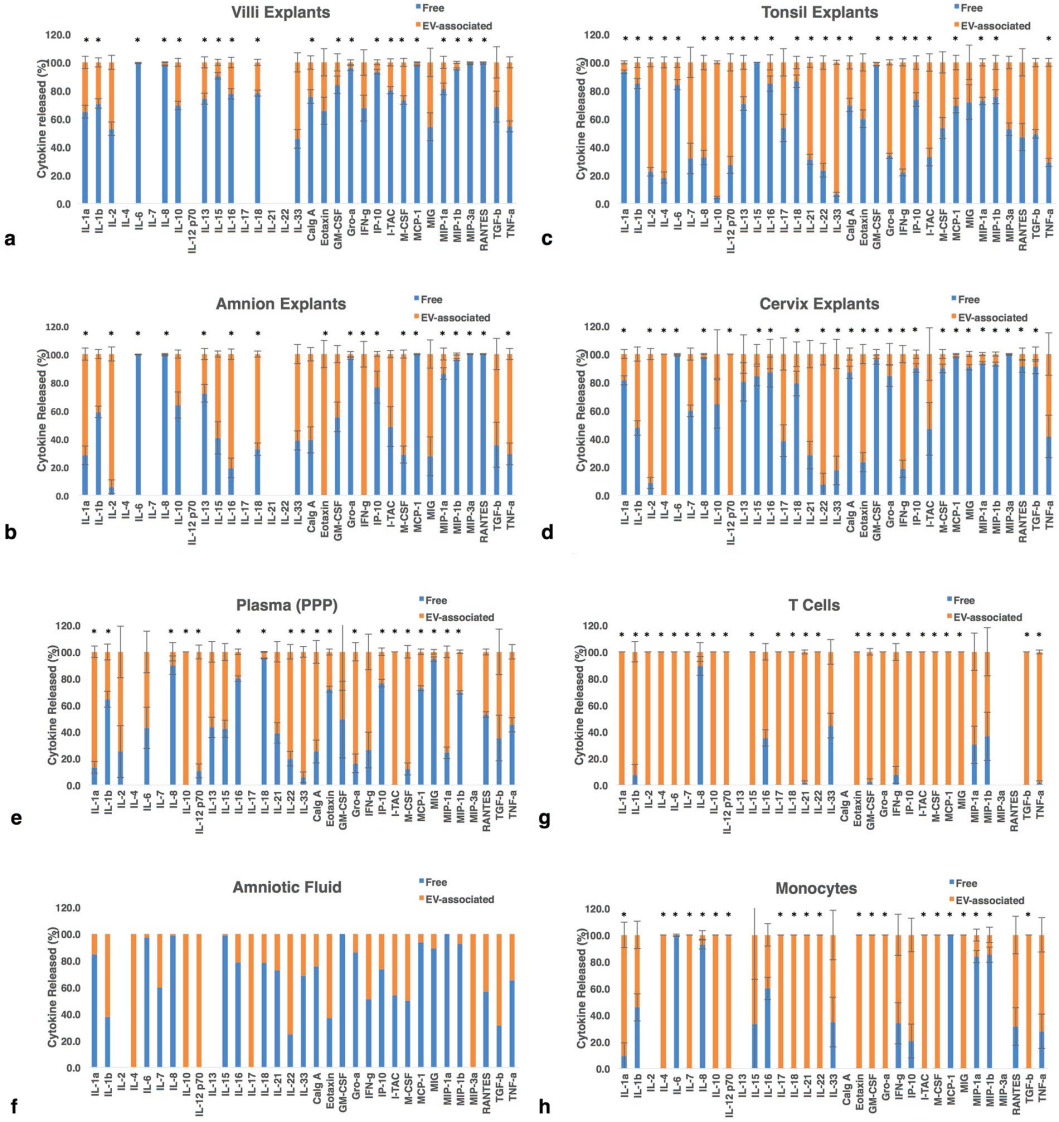
Free and EV-associated cytokines released by tissue explants. Cytokines in free form and EV-associated are expressed as percent of total cytokine released ± SEM. Blue bars: cytokine in “free” form, orange: EV-associated. Note that the same cytokines can be predominantly in free form or in EV-associated form depending on the biological system. (**a**) Placental villous explants: cumulative amounts of products released over 7 days, n = 10; (**b**) Amnion explants: amounts released over 7 days, n = 10; (**c**) Tonsillar explants: amounts released over 9 days, n = 5; (**d**) Cervical explants: amounts released over 9 days, n = 6; (**e**) PPP from healthy donors, n = 52; (**f**) Amniotic fluid from 3 donors; (**g**) T cells: amounts released over one day, n = 6; (**h**) Monocytes: amounts released over one day, n = 6. *Indicates significant difference p < 0.05 between free and EV- associated cytokines. Cumulative amounts are the sum of measurements made at day 3, 6, and 9 for tonsil and cervix, and day 1, 4, and 7 for villi and amnion explants.

We evaluated the absolute (Supplementary Table 2) and relative (Fig. 1) amounts of each cytokine in the free (soluble) fraction and in the EV fraction for each system. Distribution of cytokines between free and EV-associated form was to a large extent a characteristic of a system, rather than of the cytokines being secreted by the same pathway in all systems. In one system, a cytokine was released mostly in a soluble form, whereas in another system the same cytokine was released in EV-associated form (Fig. 1a–h).

In general, placental villous tissue preferentially secreted cytokines in free (soluble) form: 26 cytokines were found more in soluble form (>50% of total) and only 1 cytokine, IL-33, was predominantly EV-associated (Fig. 1a). Over 90% of IL-6, IL-8, IL-15, GRO-α, IP-10, MCP-1, MIP-1β, MIP-3α and RANTES were secreted in solution by placental villous explants. In contrast, T cells and monocytes produced cytokines more in EV-associated form than in free form (Fig. 1g,h).

The other studied tissues and body fluids displayed a more heterogeneous mixture of free and EV associated cytokines (Fig. 1b–f).

Analysis of individual cytokines across the various systems revealed that 9 cytokines, IL-6, IL-8, IL-13, IL-16, IP-10, MCP-1, MIP-1α, MIP-1β, and MIP-3α, were more often found in free form. Eleven cytokines, IL-2, IL-4, IL-12p70, IL-17, IL-21, IL-22, IL-33, IFN-γ, ITAC, TGF-β, and TNF-α, were found in greater levels in EVs than in the free form in most systems; 13 cytokines were relatively evenly split between two forms, being present more in EV in some systems and more in soluble form in other systems.

Amnion seems to release cytokines somewhat differently from other studied tissues. While in other tissues, IL-1α, IL-15, IL-16, IL-18, Calgranulin A, GM-CSF, M-CSF, and MIG were released predominantly in free form, for amnion tissues they were predominantly associated with EVs.

We found that the pattern of free and EV-associated cytokines released by explants during culture is relatively stable. For example, tonsil explants continued to release cytokines in similar ratios at days 3, 6, and 9 (Supplementary Figure 1).

### Cytokines associated with EVs: surface-bound vs. encapsulated

**Figure 2:**
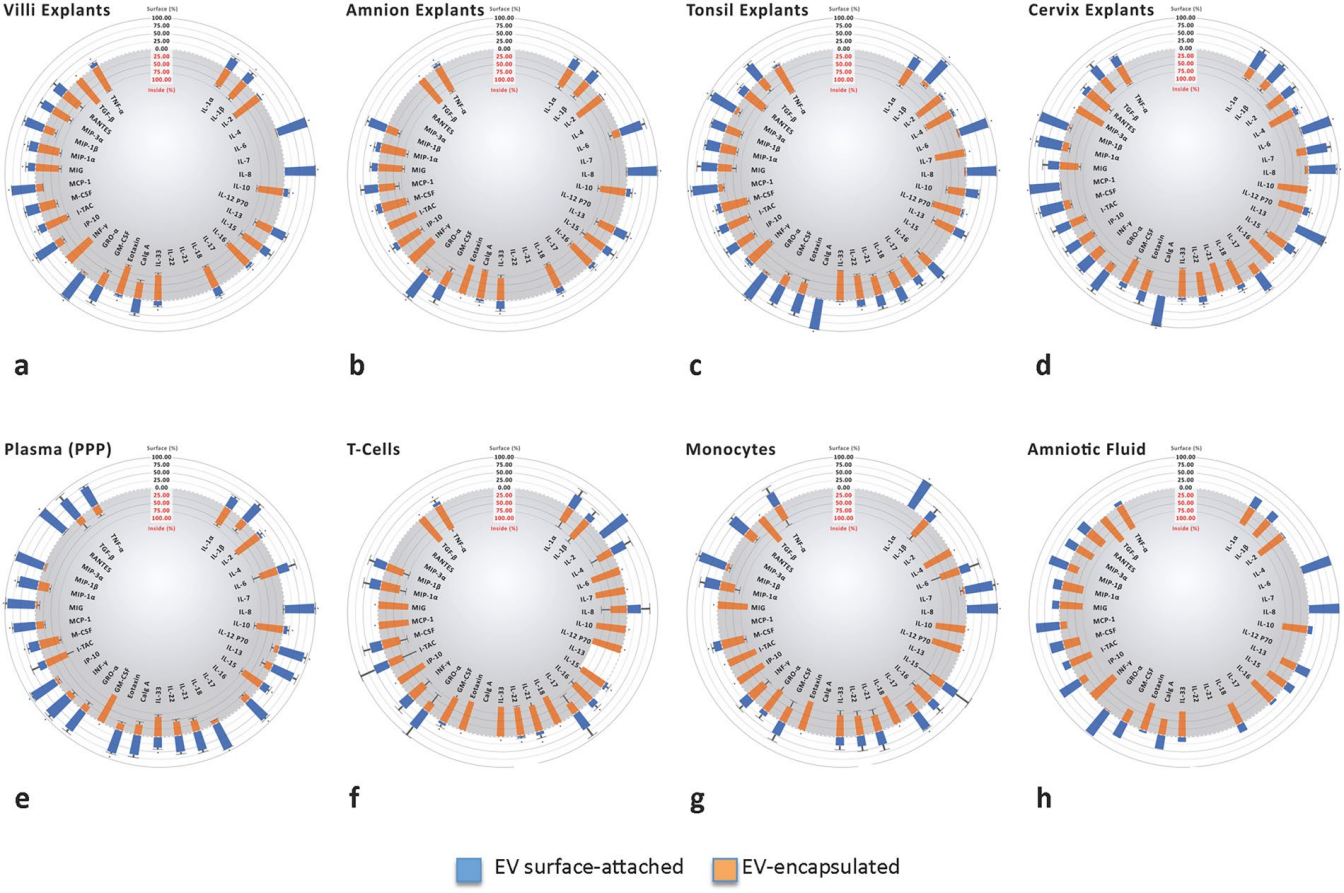
Distribution of cytokines between the surface and the inner volume of EVs. Fractions of total EV-associated cytokines ± SEM. Samples collected as in Fig. 1. (**a**) Placental villous explants n = 10; (**b**) Amnion explants, n = 10; (**c**) Tonsillar explants n = 5; (**d**) Cervical explants n = 6; (**e**) PPP from healthy donors n = 52; (**f**) Amniotic fluid from 3 donors (**g**) T cells n = 6; (**h**) Monocytes n = 6. *Indicates significant difference p < 0.05 between surface and encapsulated cytokines.

For each of the 33 cytokines in all eight systems we evaluated the cytokine fraction that was bound to the EV surface or EV-encapsulated (Fig. 2a–h). In the system of placental villous explants 7 cytokines, IL-6, IL-8, GM-CSF, GRO-α, IP-10, MCP-1, MIP-1β, were found predominantly (>50% of total) on the surface of EVs, whereas 20 cytokines were more often enclosed within EVs (Fig. 2a). In amnion explants 4 cytokines, IL-6, IL-8, GRO-α, and MIP-1β were predominantly associated with the surface of EVs, whereas 21 cytokines were predominantly inside (Fig. 2b).

EV-associated cytokines released by tonsillar and cervical explants were distributed more evenly between the surface and interior of EVs (Fig. 2c,d). Among EV-associated cytokines released by cervical explants 13 were predominantly on the surface and 19 inside EVs (Fig. 2d). However, only 6 EV surface associated cytokines released by tonsillar and cervical tissues were the same for both systems: IL-1β, IL-6, IL-8, Calgranulin A, GRO-α and MCP-1.

Platelet poor plasma displayed the greatest fraction of EV-associated cytokines on the EV surface: 20 cytokines were more on the surface and 9 were more inside EVs (Fig. 2e), the former included IL-12, IL-18, IL-21, IL-22, eotaxin, MIG, and TNFα that in other systems were encapsulated.

In contrast to plasma, in amniotic fluid out of all EV-associated cytokines only two, IL-17 and IFN-γ, were preferentially on the surface of EVs and only small fractions of another three, IL-1α, IL-22, and ITAC, were present on the surface of EVs; whereas 27 other cytokines were almost exclusively inside EVs (Fig. 2f).

For T cells (Fig. 2g) and monocytes (Fig. 2h) fewer cytokines were on the surface of EVs, whereas for both types of cells EV-associated cytokines were predominantly inside the vesicles (25 and 20 respectively).

When comparing EV-associated cytokines released by different tissues/cells/body fluids, IL-2, IL-4, IL-10, IL-12, IL-15, IL-16, IL-18, IL-21, IL-22, IL-33, Eotaxin, IP-10, ITAC, M-CSF, MIG, MIP-3α, TGF-β, and TNF-α were preferentially encapsulated in EVs, whereas IL-8, IL-17, and GRO-α were found to be greater on the surface of EVs in the majority of systems (≥75%). Among the EV-associated cytokines, IL-6 release is uniquely selective, as it was mostly bound to the EV surface when released by tissues whereas it was mostly found inside EVs in body fluids or released by cultured immune cells. Also, the rates of EV encapsulation for some cytokines were unique for particular tissues: IL-13, IFNγ, and M-CSF were higher on the EV surface only in cervix, IL-16 and eotaxin were higher on the EV surface only in tonsil, IP-10 was higher on the EV surface only in villi, and MCP-1 was higher inside EVs only for amnion explants.

We investigated whether the EV-encapsulated cytokines were more stable than free cytokines. In experiments with tonsil and placental villous explants, we took samples of cell-free conditioned medium (containing both free and EV-associated cytokines) and incubated them for 2 days at 37 °C and then compared the concentration of cytokines with those measured immediately. The amount of free cytokines was significantly decreased in the conditioned medium by ~30% (70.2 ± 4.6% of the original amount remained (p = 0.01, n = 6)), while the amount of EV encapsulated cytokines was not different from the initial amount (88.8 ± 8.9% of original concentrations (p = 0.76, n = 6)). Least stable in the free form were IL-1β, IL-10, IL-13, IL-15, IFNγ, IP-10, M-CSF, MIP-1α, MIP-1β, MIP-3α, and RANTES and TNF-α, while these and all other EV-encapsulated cytokines were protected.

In an attempt to address the nature of vesicles involved in cytokine encapsulation, we used three pharmacological inhibitors: ketotifen and manumycin A to inhibit exosomes^2,3^, and imipramine to inhibit ectosomes^4^. The strongest effect was obtained with manumycin A which decreased the amount of several EV-associated cytokines, namely IL-1α, IL-1β, IL-6, GRO-α, MCP-1, MIP-1α, MIP-1β, and MIP-3α, by 55–90%, consistent with their association with exosomes. Ketotifen and imipramine had less pronounced effects.

### Activation changes EV-cytokine association

**Figure 3:**
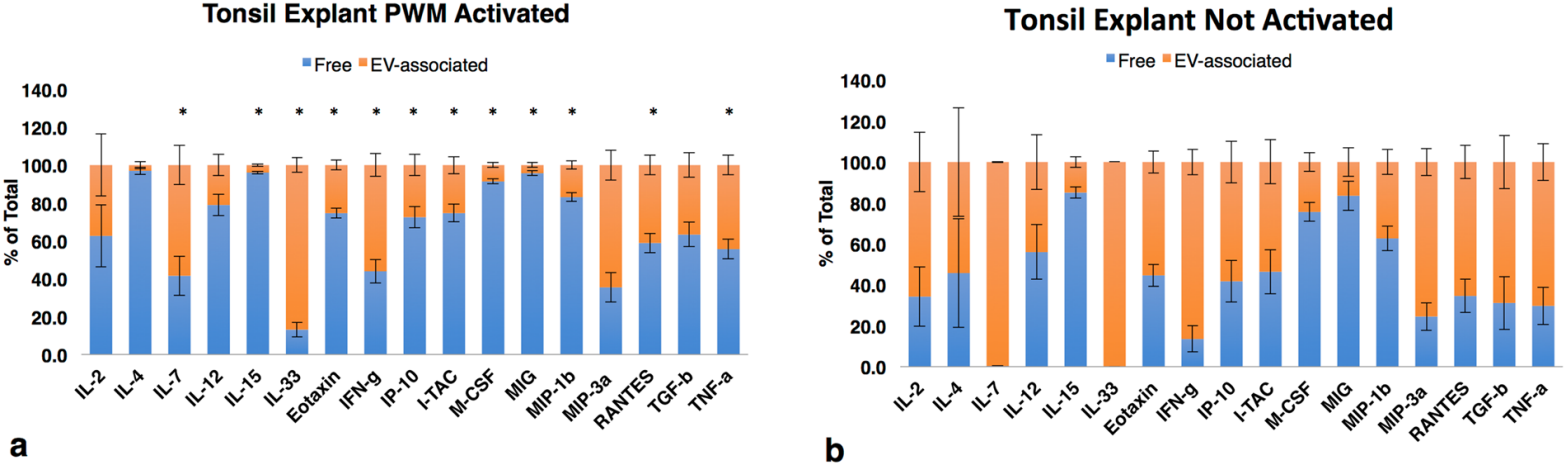
Cytokine distributions between EV-associated and free forms in stimulated tonsils. Tonsil explants with or without stimulation by pokeweed mitogen for 3 days. Supernatants free of EV and EVs lysed with 1% Triton-X. Free and EV-associated cytokines are expressed as percent of total ± SEM. Blue bars: “free” cytokines, orange: EV-associated, n = 4. Cytokines released by tonsil explants: (**a)** stimulated with pokeweed mitogen; (**b)** without stimulation. *Indicates significant difference p < 0.05 in cytokine distribution between PWM-activated and controls.

We investigated whether in the same system this pattern may be modulated. We compared the release of EVs by untreated and activated tonsillar explants. Activation with pokeweed mitogen resulted in drastic changes in the pattern of release of cytokines (Fig. 3a,b): For IL-7, IL-15, IL-33, Eotaxin, IFN-γ, IP-10, ITAC, M-CSF, MIG, MIP-1β, RANTES, and TNF-α there was a statistically significant (p < 0.05) shift toward a more soluble and less EV-associated pattern of cytokine secretion.

**Figure 4:**
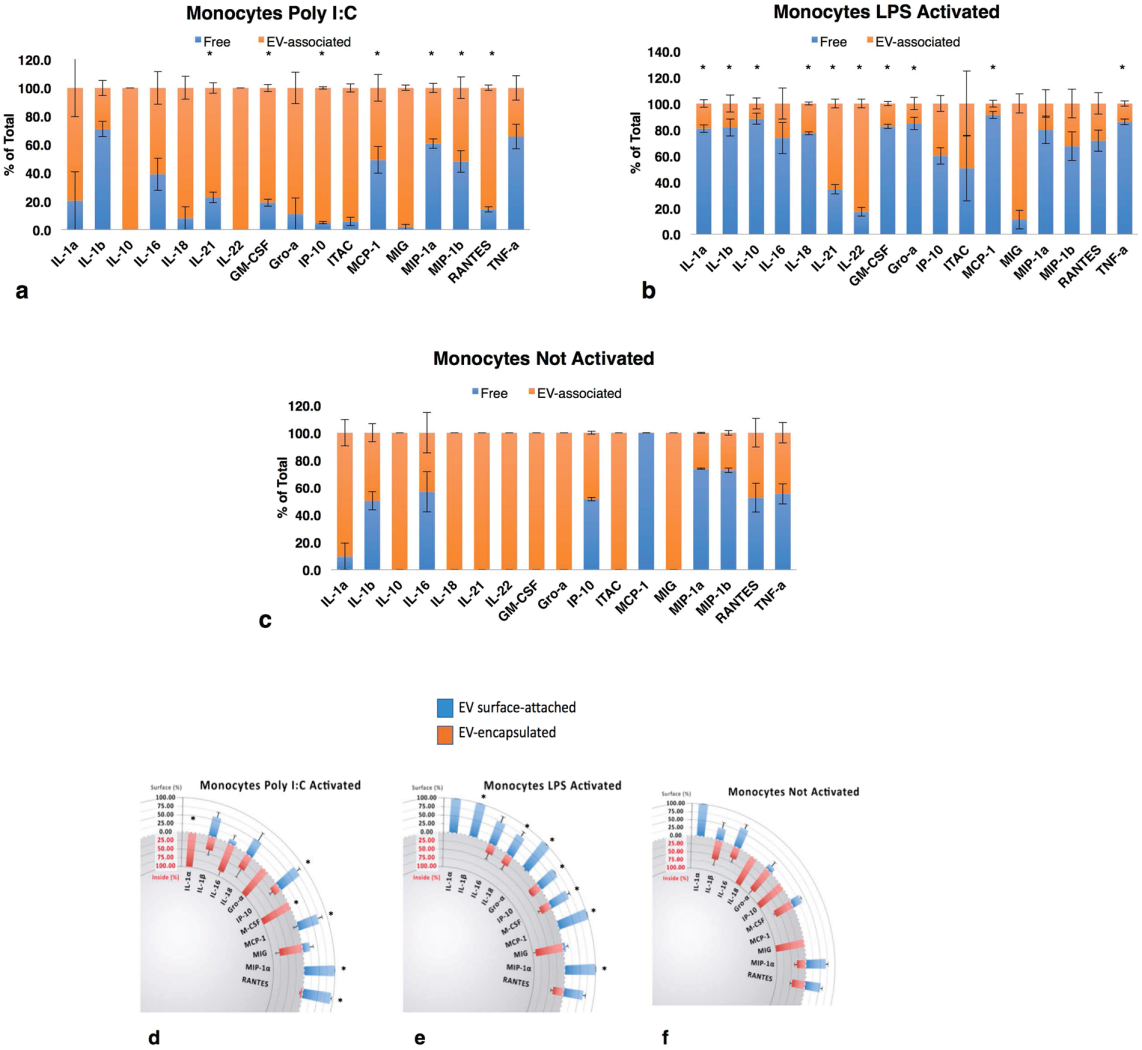
Cytokine distributions between EV-associated and free forms in stimulated monocytes. Monocytes were cultured without stimuli **(c)** or stimulated by poly I:C (**a**) or LPS (**b**) Free and EV-associated cytokines are expressed as percent of total ± SEM. Blue bars: “free” cytokines, orange: EV-associated. Distribution between encapsulated and surface attached selected cytokines which changed with stimulation a and b. Stimulation with poly I:C (**d**), LPS (**e**) and control (**f**) n = 3. *Indicates significant difference p < 0.05 in cytokine distribution between activated and control monocytes.

We investigated whether there was a change in cytokine secretion pattern of monocytes activated with two different stimuli, either LPS or Poly I:C (Fig. 4a,b,c). Activated monocytes release slightly more EVs (1.6 ± 0.2 compared to control (p = 0.06, n = 3)), however, the pattern of cytokine association with EVs was changed significantly and was dependent on the stimuli. Monocytes activated by Poly I:C displayed shifts toward higher amounts of soluble cytokines especially for IL-21 and GM-CSF, and shifts toward more EV-associated cytokines for IP-10, MCP-1, MIP-1α, MIP-1β and RANTES (p < 0.05). Activation of monocytes with LPS resulted in a statistically significant (p < 0.05) decrease in the fraction of EV-associated IL-1α, IL-1β, IL-10, IL-18, IL-21, IL-22, GM-CSF, Gro-α, and TNF-α. A similar trend was observed for several other cytokines (Fig. 4). In contrast, MCP-1 shifted to more EV-associated form (p < 0.05).

Not only the distribution of cytokines between EVs and free soluble form is changed upon monocyte activation, but the pattern of cytokine EV encapsulation versus surface association was modified as well, again in a stimulus-specific way (Fig. 4d,e,f). Monocytes activated by Poly I:C displayed shifts toward higher amounts of EV surface cytokines, which were significant (p < 0.05) for IP-10, MCP-1, MIP-1α and RANTES, and shifts toward more EV-encapsulated cytokines for IL-1α and M-CSF (p < 0.05). Activation of monocytes with LPS resulted in the general shifts in cytokine release toward more EV surface bound form, especially for IL-1β, IL-18, Gro-α, IP-10, M-CSF, MCP-1, and MIP-1α (p < 0.05).

Thus, in the same system, activation resulted in dramatic changes in the pattern of cytokines’ release and their encapsulation in EVs.

### EV-encapsulated cytokines trigger responses in sensitive cells

**Figure 5:**
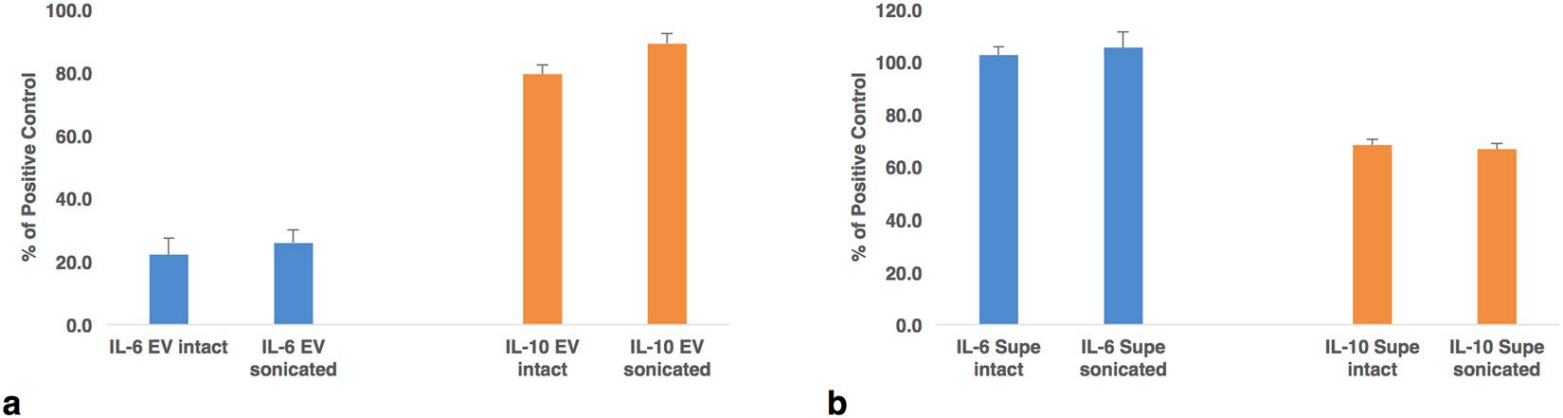
Biological activity of EV-encapsulated cytokines. The average levels of metabolic activity ± SEM compared to that of positive control cells in optimal growth medium as measured by MTT. IL-6 activity was measured in TF-1 cells; IL-10 activity in MC/9 cells. (**a**) Intact or sonicated EVs from PPP assayed for IL-6 activity (n = 3) and from activated monocytes for IL-10 activity (n = 3). (**b**) Indicator cells incubated with intact or sonicated supernatants from EVs from tonsil explants assayed for IL-6 (n = 5) and IL-10 (n = 4).

With the discovery of so many cytokines associated with EVs, we investigated whether these cytokine-carrying EVs are biologically active. To answer this question, we incubated EV-associated cytokines with indicator cell lines TF-1 and MC/9 that are dependent on particular cytokines for their proliferation^5^. First, we tested EVs isolated from plasma that carry IL-6 more on the surface of EVs than inside (66.7 ± 1.9% on the surface and 8.3 ± 1.4% inside EVs). We found that these EVs were active in eliciting responses from sensitive cells, and the response to the same EVs that were sonicated to release the internal cytokines was not significantly different from the response to the intact EVs (n = 3, p = 0.600) (Fig. 5a). Similar results (p = 0.708, n = 5) were obtained (Fig. 5b) when the sonication experiments were performed with the entire medium rather than with isolated EVs, this time using supernatants from tonsil cultures which contain 69.3 ± 3.9% of total IL-6 in free form and 30.7 ± 3.9% EV-associated.

Similar experiments were conducted with intact and sonicated EVs from activated monocyte cultures which have 100 ± 0% of IL-10 encapsulated in EVs and none on the EV surface. Responses to both intact and sonicated EVs were similar (n = 3, p = 0.094) (Fig. 5a). Likewise in experiments with intact and sonicated supernatants from tonsil cultures, in which 4.6 ± 1.9% of IL-10 was in free form and 95.4 ± 3.9% was EV-associated (with 63.3 ± 3.9% encapsulated), there was not a significant difference in response (p = 0.676, n = 4) (Fig. 5b). These experiments provided evidence that EVs that carry cytokines are biologically active and the encapsulated cytokines contribute to their activity.

## Discussion

Extracellular vesicles (EVs) represent a diverse family of lipidic particles pinched off plasma membranes or released from multivesicular bodies (exososmes) as reviewed in^6^. EVs have been shown to alter characteristics of cells exposed to them^7–9^ thus constituting a third system of cell-cell communications, complementing cell-cell contacts and communication by soluble factors, like cytokines. Although cytokines are generally thought to exert biologic influence as soluble molecules, several cytokines have been reported to be associated with extracellular vesicles, for example, a membrane bound form of TNF-alpha from individuals with osteoarthritis^10^, chemokines associated with lipid rafts^11^, multiple cytokines inside human umbilical cord mesenchymal stem cell EVs^12^, or cytokines, such as the IL-1 family, which lack a signal peptide for secretion by the classical pathway^13^. Additionally, it was shown that the amount of EV-associated cytokines was increased in HIV-infection^14^. Whether association with EVs reflects the chemical property of particular cytokines or whether association with EVs is a general regulated physiologic process remains to be understood.

Here we undertook a first comprehensive study of cytokine association with EVs in an attempt to answer the following questions: (i) how general is the phenomenon of cytokine association with EVs? (ii) are only particular cytokines associated with EVs? (iii) can a cytokine released in association with EVs in one system get released as a free (soluble) molecule in another? (iii) is the association of cytokines with EVs a regulated process that can be modulated? (iv) are cytokines encapsulated in EVs rather than merely attached to them? (v) can EV-encapsulated cytokines be delivered to sensitive cells and trigger a physiological response?

To answer these questions, we systematically analyzed the expression of 33 cytokines and their association with EVs in eight diverse *in vitro*, *ex vivo* and *in vivo* biological systems.

We demonstrated that being associated with EVs is not necessarily the property of a cytokine but rather is the regulated property of a system. In the eight different systems we studied, a given cytokine in one system can be released predominantly in a soluble form, while in another it can be predominantly EV-associated. Indeed, placental villous explants preferentially elaborated more cytokines in soluble form, including eight cytokines that were found over 90% in the free form. In contrast, T cells and monocytes released some of the same cytokines predominantly associated with EVs. The difference between placental villous explants and immune cells may be related to the high level of expression of cytokines by these tissue explants while T cell and monocyte suspensions that express much lower levels of cytokines may need to concentrate them in EVs rather than dissolving them in solution.

Among EV-associated cytokines IL-2, IL-4, IL-10, IL-12, IL-15, IL-16, IL-18, IL-21, IL-22, IL-33, Eotaxin, IP-10, ITAC, M-CSF, MIG, MIP-3α, TGF-β, and TNF-α were preferentially encapsulated in EVs across all systems, in some instances all EV-associated cytokine was inside EVs (for example, IL-10 in cervix, T cell and monocyte cultures, and IFN-γ in placental villous and amnion cultures as well as in amniotic fluid).

Thus, different biological systems differentially distribute the released cytokines between free and EV-associated forms. The pattern of the cytokine released is not a fixed property of the system but rather can be modulated, for example upon activation. In our experiments, the total number of EVs increased upon stimulation but only slightly probably because the total number of EVs may be the result of superimposition of EVs released by activated and non-activated cells. In general, the increased release of EVs was reported earlier and often was associated with immune activation^15,16^. Importantly, the relative fractions of free and EV-associated forms of cytokines are altered upon activation. Moreover, the two stimuli used here to activate monocytes changed the pattern of cytokine association with EVs in dramatically different patterns. In general, with some exceptions, relatively more cytokines are released in a free form upon stimulation and the association with EVs shifted from encapsulated to surface-attached forms. Whether these changes in the pattern of cytokine release occur at the single-cell level or some cells release free cytokines while others release EV-associated ones remains to be determined as all of the systems, including cultures of T cells or monocytes are comprised of populations that are heterogeneous in both activation status and phenotype. In any case these experiments show that the term “activation” may describe very diverse biological processes, in particular, in relation to cytokine encapsulation in EVs. The ability of one system to change the pattern of EV encapsulation of cytokines is another indication of the tight biological regulation of this process.

There could be multiple biologic meanings to loading EVs with cytokines. It was suggested that EV-entrapment is a mechanism to dispose of products when they are over-produced^9^ simultaneously protecting the releasing cell from an autocrine effect. More probable is that EVs protect cytokines from environmental degradation. Indeed, EV-entrapped cytokines are protected from trypsin digestion. Also, in a more physiological environment, in the medium conditioned by tissue explants, free cytokines started to degrade in two days at 37 °C, while EV-encapsulated ones were largely protected. Also, EV-encapsulation may facilitate cytokine delivery and targeting to distant target cells. This targeting could be mediated by binding of EV-surface cytokines to cells that express specific cytokine receptors. Also, sialic-acid binding immunoglobulin lectins^17^, C-type lectins^18^, lactadherin^19^, membrane receptors like MHC I and II^20^, transferrin receptors^21^, and tetraspanins^22^ as well as viral proteins^23^ have been suggested as promoting targeting of EVs. In general, encapsulating cytokines in EVs may be a mechanism whereby the cytokine expressing cell could expand its sphere of influence to concentrate cytokines at the surface of other cells that might not otherwise be targeted by cytokines in solution.

Such a targeting would require that EV-associated cytokines are biologically active. Here we provide evidence for such activity using reporter cell lines that need particular cytokines to proliferate. We found that these cells responded to EVs containing IL-6 and IL-10 even when all of the cytokine is encapsulated. Our sonication experiments indicated that the biological activity of the EV-encapsulated cytokines was the same whether we released the cytokines or provided them in EVs. In our model experiments EVs play a role of passive carriers releasing cytokines upon contacting cell surface. *In vivo* EVs may be delivered to selective target cells that respond to these EVs depending what cytokine receptors these cells express and what cytokines are associated with EVs. This complex aspect of the *in vivo* situation is not reflected in the *in vitro* experiments with cell lines.

Since the cellular receptors for both IL-6 and IL-10 as well as for most cytokines, are on the cell surface^24^ mechanisms by which EV-encapsulated cytokines trigger biological effects should include the release of the entrapped cytokines when EVs interact with the cell surface. Such a mechanism has been demonstrated with other lipid vesicles which become leaky upon their contact with plasma membrane^25,26^ or in the process of fusion^27^. Similarly, a stimulus-dependent mechanism was suggested for cytokine liberation from cell-free eosinophil granules^28^.

In principle, the release of cytokines at the surface of a cell should be more efficient than depositing free molecules into the extracellular space since the release of the vesicle cargo in the close vicinity of target cells would create a high surface concentration even with a small quantity of released molecules. Therefore, while the biological relevance of EV-associated cytokines whose concentration is comparable to those in a free form (e.g., IL-1α (plasma, amnion, cervix), IL-10 (tonsil, cervix), IL-16 (amnion, T cells, monocytes), IL-17 (tonsil), eotaxin (villi, tonsil), Gro-α (tonsil), MCP (tonsil), MIP-3α (tonsil)) is obvious, we cannot exclude that even when the amount of cytokines associated with EVs is small (e.g IL-6, IL-8, IP-10, MIP-1α, MIP-1β) they play an important role as they are delivered to the cell surface receptor rather than dissipated in a large volume.

In spite of a significant fraction of cytokines being encapsulated in EVs, these cytokines are not detected by standard target cell-free cytokine assays, such as ELISA or other multiplexed immunoassays since they are hidden from the cytokine-specific detection antibodies by the EV membrane. In our work, these cytokines were released by detergent or sonication. None of the conventional protocols for cytokine measurements include these procedures. Therefore, the interpretations of the roles of these cytokines in health and disease based on these standard assays should be now reconsidered. For example, by traditional assays ITAC produced by LPS activated monocytes would be 2-fold higher than Poly I:C activated (161 versus 81 pg/ml), but when EV encapsulated cytokines are taken into account, LPS monocytes actually produce slightly less ITAC (238 versus 283 pg/ml).

Our study has several limitations: We did not separate EVs into different fractions by size or by the presence of particular surface molecules to allow analysis of these fractions separately. Undoubtedly, patterns would emerge to show preferential packaging of particular cytokines into particular EVs. In an attempt to reveal these patterns, we used pharmacologic inhibitors of exosomes (ketotifen and manumycin A^2,3^) and of ectosomes (imipramine^4^). The strongest inhibition of several cytokines (55–90%) was obtained with manumycin A. However, it would be premature to conclude that these cytokines are associated with exosomes since inhibition by the other two inhibitors was less pronounced and affected different cytokines. Also, not all of these inhibitors are 100% specific^2–4^, and moreover, in different cells different cytokines may be encapsulated in different vesicles. Another limitation is that we do not know the stability of different types of vesicles that may affect their ability to retain enclosed cytokines. It was reported that exosomes are relatively stable^29,30^, however the stability of other vesicles has not been well studied.

Regardless of cytokine segregation in different EVs and their biogenesis, EVs seem to protect the encapsulated cytokines as shown by their stability, compared to free cytokines, when kept in conditioned medium for 2 days at 37 °C. This may be an important biological meaning of cytokine encapsulation while EV surface associated cytokines may contribute to addressing EVs to particular distant cells that express appropriate cytokine receptors.

In summary, our work is the first systematic study on cytokine association with EVs in diverse biological systems that showed that (i) cytokine encapsulation into EVs seems to be a general biological phenomenon observed in different *in vitro*, *ex vivo* and *in vivo* systems; (ii) although some cytokines are preferentially released in EVs and others in a free form, it seems that any given cytokine can be encapsulated into EVs; (iii) the same cytokine can be released in association with EVs in one biological system while in another it can be released as a free (soluble) molecule; (iv) in the same biological system, the pattern of cytokine encapsulation into EVs is dramatically changed by activation; (v) EVs that encapsulate cytokines can deliver them to sensitive cells and trigger their physiological response.

The release of cytokines either in a free or EV-associated form might reflect an adaptation to specific physiological needs, in particular whether these cytokines are needed to act near the secreting cell or at a distance. In support of this concept, tissue explants where cells were sustained in close proximity to the cells contained in their *in vivo* neighborhoods, tended to release more of their cytokines in a soluble form than were found in T cell or monocyte cell suspensions or in plasma where a greater proportion of cytokines were found associated with EVs. A system of EV-encapsulated cytokines as described above may represent an important system of cell-cell communication in health and disease and may serve as a new therapeutic target.

## Methods

### Sample preparation and storage

Placental tissues (the placenta and fetal membranes) from women who delivered at term without labor (n = 10) were obtained at the Detroit Medical Center, Wayne State University, and the Perinatology Research Branch, an intramural program of the *Eunice Kennedy Shriver* National Institute of Child Health and Human Development, National Institutes of Health, US Department of Health and Human Services (NICHD/NIH/DHHS) (Detroit, MI, USA). The collection and utilization of biological materials for research purposes were approved by the Institutional Review Boards of these institutions. All participating women provided written informed consent. Immediately after delivery, placental tissues were transported to the laboratory and three random samples from the placental villi were collected using a metal grid and the Random Position Generator DICE software (Perinatology Research Branch, Detroit, MI, USA). In addition, the amnion was gently separated from the chorion of the fetal membranes. Samples from the placental villi and amnion were placed in individual 50 mL tubes containing DMEM and shipped overnight to NIH on cold packs. Upon receipt, villi were sectioned into strips of approximately 2 mm × 6 mm, washed 3 times in PBS and cultured on Gelfoam adsorbable collagen sponges (Pharmacia and Upjohn) at the air-liquid interface, as has been described for other tissues^31^ in 0.1 μm filtered phenol red free DMEM (Thermo Fisher) supplemented with 5% characterized, charcoal stripped FBS (Hyclone), 50 μg/ml gentamicin and 2.5 μg/ml Amphotericin B (Thermo Fisher) at 37 °C, 5% CO_2_. Amniotic membrane was sectioned into 3 × 3 mm pieces, washed three times with PBS, and cultured in same medium. Equivalent masses were cultured in three wells of a six-well plate for each donor. Medium was collected and changed at days 1, 4, and 7 after initiation. Medium was centrifuged at 400 × g for 5 minutes to remove cells and frozen at −80 °C.

Tonsillar tissue (n = 5) was obtained from routine tonsillectomies performed at the Children’s National Medical Center in Washington DC. Cervical tissue (n = 6) from routine hysterectomy was received through the National Disease Research Interchange (NDRI, Philadelphia, PA). All tissues were anonymous pathological samples obtained according to an Institutional Review Board approved protocol. Healthy tonsil tissue and mucosal areas of both ecto- and endo-cervix were dissected as previously described^31^. Briefly, tissue was dissected into 2 mm × 2 mm blocks and cultured on collagen sponges at the air liquid interface, with a minimum of 18 blocks per condition, in RPMI 1640 (Thermo Fisher) supplemented with 15% FBS (Gemini Bioproducts), 100 μM Modified Eagle’s medium (MEM)-nonessential amino acids, 1 mM sodium pyruvate, 50 μg/ml gentamicin, 2.5 μg/ml Amphotericin B (all from Thermo Fisher). Medium was collected and changed at day 3, 6, and 9. Collected medium was centrifuged at 400 × g for 5 minutes to remove cells and aliquots were frozen at −80 °C. Activated tonsil cultures were incubated with 2.5 μg/ml of pokeweed mitogen (PWM, Sigma) for 3 days and medium was collected as above.

Platelet poor plasma (PPP) was received from the Laboratory of Atherothrombosis at the Moscow University of Medicine and Dentistry under an IRB-approved protocol from 52 donors. Briefly, peripheral blood was collected from healthy volunteers with risk factors of coronary artery disease (mild hypertension, obesity, dyslipidemia, smoking) into vacuum tubes with sodium citrate and centrifuged at 3,000 × g for 15 minutes for removal of cells and recovery of plasma. The plasma was again centrifuged at 3,000 × g for 15 minutes to remove platelets. Aliquots were frozen at −80 °C and later shipped to NIH on dry ice.

T cells (n = 6) and monocytes (n = 6) were harvested from whole blood obtained with written informed consent at Case Western Reserve University/University Hospitals Cleveland Medical Center, OH under approval of the Institutional Review Board of University Hospitals. Following preparation of peripheral blood mononuclear cells (PBMCs) by Ficoll-Hypaque (GE Healthcare) density sedimentation, T cells were enriched using magnetic bead negative selection (Miltenyi). Monocytes were enriched from whole blood using RosetteSep (STEMCELL) purification followed by CD61 + platelet depletion (Miltenyi). T cell purity was 97% ± 1.17 and monocyte purity was 78% ± 2.26. Cells were cultured overnight in RPMI medium supplemented with 10% heat-inactivated fetal bovine serum, 1% penicillin/streptomycin, and 1% L-glutamine at 37 °C and 5% CO_2_. Culture supernatants were collected, centrifuged at 2000 × g for 15 minutes to remove cells and aliquots were frozen at −80 °C and later shipped to NIH on dry ice. For activated monocyte cultures, cells were incubated with lipopolysaccharide (LPS, Sigma) at 100 ng/ml or Poly I:C (Invivogen) at 25 μg/ml for 24 hours and medium was collected as above.

Amniotic fluid was collected by transabdominal, ultrasound-guided amniocentesis from women enrolled at Hutzel Women’s Hospital (Detroit, MI). All women provided written informed consent prior to the collection of biological samples. The utilization of samples for research purposes was approved by the Institutional Review Boards of Wayne State University and the National Institute of Child Health and Human Development (NICHD/NIH/DHHS). Amniotic fluid not required for clinical assessment was centrifuged for 10 minutes at 4 °C at 1300 × g for 10 minutes shortly after the amniocentesis, and the supernatant was aliquoted and stored at −80 °C. Fluid from 3 donors was pooled and aliquots were frozen at −80 °C and shipped to NIH on dry ice.

### Preparation of EV fractions

All samples collected above were split into multiple fractions. One aliquot was kept untreated, another portion was treated with ExoQuick (SBI) for plasma or Exoquick TC for all other fluids according to manufacturer’s protocols. Briefly, Exoquick was added to plasma without addition of thrombin at a ratio of 63ul of Exoquick to 250 μl plasma and refrigerated for 30 minutes at 4 °C. For all other samples, ExoQuick TC was added to supernatants at a ratio of 100 μl of ExoQuick TC to 500 μl of sample and refrigerated overnight at 4 °C. ExoQuick/sample mixtures were centrifuged at 1500 × g for 30 minutes to pellet EVs. Supernatant was collected and saved for cytokine measurement of EV-free supernatant. The pellet was centrifuged again at 1500 × g for 5 minutes and all traces of fluid were removed resulting in an EV enriched preparation. The pellet was resuspended in 1X PBS in the original volume and saved for measurement of cytokines on intact and lysed EVs. In most instances, these fractions were stored at 4 °C and run within 24 hours. However, T cell and monocyte fractions collected at Case Western Reserve University were stored at −80 °C until shipment to NIH on dry ice. For experiments where EV fractions were added to cell cultures, EV contents were released by sonication for 3 x 10 minutes in an ultrasonic water bath^32^. Release of cytokines from EVs by sonication was compared to detergent lysis using EVs from tonsillar, villous, and amnion explants. Sonicated EVs released on average 98.9 ± 6.4% of that released by detergent lysis. The only exception was Calgranulin A, the amount of which cannot be correctly measured in the presence of Triton X (see below).

EVs from tonsil and placental villous explants were isolated by ExoQuick procedures and treated with 0.25% trypsin-EDTA (Thermo Fisher) for 20 minutes at 37 °C to remove EV surface bound cytokines. Trypsin digestion was halted by addition of serum and EVs were isolated again by ExoQuick, followed by cytokine evaluation of intact and lysed EVs.

To verify that free cytokines are not co-precipitated with EVs when using ExoQuick, we spiked complete culture medium with recombinant cytokines of known quantities for all proteins measured. This spiked medium was treated with ExoQuick, and supernatant and EV fractions were collected and cytokines were measured in these fractions. We also verified that EVs from FBS did not contribute to our cytokine measurements. Next, the same recombinant protein spiked culture medium was incubated with EVs from Jurkat cells for one hour at 37 °C and treated with ExoQuick. Supernatant and EV fractions were analyzed for cytokine content and compared to levels of Jurkat EVs only (which contained low levels of only a few cytokines).

LPS activated monocytes were treated with 1 μM and 10 μM ketotifen, manumycin A, imipramine (Sigma). Medium samples were collected after 24 hours and analyzed for EV-associated cytokines.

### NanoSight measurement of EV

Aliquots of samples (not treated with ExoQuick) were diluted and characterized with Nanoparticle Tracking Analysis software using a NanoSight NS300 (Malvern), which uses light scattering and Brownian motion to obtain particle size distributions and concentrations. Briefly, samples were diluted 1:10 or 1:100 in particle-free PBS and placed in the analyzer. Three representative samples for each system were analyzed by 2 video captures of 60 seconds each to generate averaged concentrations of EV/ml ± SEM, mean ± SEM particle size (nm), mode (most commonly found particle size) ± SEM of particle size (nm), and D90 (the size above which 90% of particles are contained) ± SEM (nm). Samples from tissue explants were evaluated on day 3 (accumulation of EVs from day 1–3 of culture) for tonsillar and cervical explants, and day 4 (accumulation of EVs from day 2–4 of culture) for villi and amnion explants. Samples from T cells and monocytes were evaluated at day 1 of culture.

### Cytokine measurement

We previously developed an in-house multiplexed bead-based assay for measurement of the following 33 cytokines: IL-1α, IL-1β, IL-2, IL-4, IL-6, IL-7, IL-8, IL-10, IL-12p70, IL-13, IL-15, IL-16, IL-17, IL-18, IL-21, IL-22, IL-33, Calgranulin A (S100A8), Eotaxin (CCL11), granulocyte-macrophage colony-stimulating factor (GM-CSF), growth-regulated alpha (GRO-α or CXCL1), interferon-γ (IFN-γ), interferon-γ-induced protein (IP-10 or CXCL10), interferon-inducible T-cell alpha chemoattractant (ITAC or CXCL11), macrophage colony-stimulating factor (M-CSF), monocyte chemoattractant protein-1 (MCP-1 or CCL2), monokine induced by IFN-γ (MIG or CXCL9), macrophage inflammatory protein-1α (MIP-1α or CCL3), MIP-1β (CCL4), MIP-3α (CCL20), regulated on activation normally T-cell expressed and secreted (RANTES or CCL5), transforming growth factor-β (TGF-β), and tumor necrosis factor-α (TNF-α) as described previously with slight modifications^33,34^. All antibody pairs and cytokine standards were purchased from R&D Systems except those for IL-4 (Biolegend) and IL-21 (eBioscience). Magnetic beads (Luminex) with 33 distinct spectral signatures (regions) were coupled to cytokine specific capture antibodies according to manufacturer’s recommendations and stored at 4 °C. All cytokine pairs were verified to be free of cross reactivity. Standards and samples were diluted in assay buffer (1X PBS with 20 mM Tris-HCl, 1% each normal mouse and goat serum (Gemini Bioproducts) and 0.05% Tween 20) and combined with bead mixtures and incubated overnight at 4 °C. Intact EV samples and lysed EV samples to which Triton X was added at final concentration of 1% were run in separate wells. Plates were washed 3 times and incubated with mixtures of polyclonal biotinylated anti-cytokine antibodies (R&D Systems) in assay buffer for 1 hour at room temperature. Plates were washed three times and incubated for 25 minutes with 16 μg/ml streptavidin-phycoerythrin (Invitrogen) in PBS. Plates were washed 3 times and beads were resuspended in PBS. Plates were read on a Luminex 200 analyzer with acquisition of a minimum of 100 beads for each region and analyzed using Bioplex Manager software (BioRad). Cytokine concentrations were determined using 5P regression algorithms. Concentrations of analytes in EV free supernatants were adjusted for dilution by ExoQuick reagent. For tissue explants, the sum of cytokines over the entire culture period (7–9 days) was calculated, T cells and monocyte cytokines were measured after 1 day in culture, and body fluids were measured directly *ex vivo*.

We evaluated the effect of detergent treatment on “free” cytokines by measuring cytokines in the supernatant fractions after Exoquick treatment (containing no EVs) with and without Triton X. We found that the difference between measuring free cytokines with or without detergent was statistically not different. The only exception was Calgranulin A, for which Triton X interfered with the assay by decreasing the apparent measurement to 43.4 ± 4.4% that of control without detergent (n = 12, p = 0.01).

### Cytokine bioassay

Cultured cell line-based cytokine driven proliferation assays were used to test bioactivity of cytokines in the EV fractions. TF-1 (ATCC) is a human erythroleukemic cell line responsive to a number of human cytokines including GM-CSF, IL-3, IL-4, IL-5, IL-6 and IL-13, as well as erythropoietin, leukemia inhibitory factor, nerve growth factor, and stem cell factor. This cell line was cultured in RPMI-1640, 10% FBS, 2mM L-glutamine, 1% sodium pyruvate, 2 ng/ml GM-CSF. We used proliferation of this cell line to measure activity of IL-6. MC/9 (ATCC) is a mouse mast cell line used to measure activity of IL-10. MC/9 is cultured in DMEM high glucose supplemented with 1.5 g/L sodium bicarbonate, 2mM L-glutamine, 50 μM 2-mercaptoethanol, 10% Rat T-STIM (Becton Dickenson), and 10% FBS. Cells were counted, washed twice in PBS, and resuspended at a concentration of 2 × 10^5^ cells/ml in culture medium without exogenous cytokine or T-STIM. Briefly, 50 μl of cells per well were added to 96 well flat bottom plates and then treated with intact or sonicated supernatants or EV fractions. Since lysed fractions are not compatible with cell culture we instead sonicated supernatant or EV fractions for 30 minutes and verified that this released EV contents (IL-6 released 83.7 ± 8.2% and IL-10 92.9 ± 6.0% that released by detergent lysis). These fractions were added at 50 μl per well, in addition to positive control wells of cells with complete growth medium and negative control wells of cells with medium not containing growth factors. Cells were incubated for 48 hours at 37 °C and 5% CO_2_. Metabolic activity was measured by an MTT (3-(4,5-dimethylthiazol-2-yl)-2,5-diphenyltetrazolium bromide) assay. MTT (Sigma) was prepared at 5 mg/ml in PBS and sterile filtered. 5 μl of MTT was added to each well and plates were incubated for 4 hours at 37 °C. Lysis buffer of 20% sodium dodecyl sulfate (SDS)/50% dimethyl formamide (DMF)(Sigma) was added at 50 μl per well and plates were incubated for 1 hour and absorbance was measured at 570 nm on Tecan Sapphire 2 using Magellan 5.0 software. Plates were read a second time after overnight incubation, to ensure all formazan crystals were completely dissolved. Results were expressed as % of positive control (cells in complete growth medium).

### Statistical Analysis

We conducted statistical analysis using JMP10 (SAS Institute). Results are represented as means ± standard error of the mean (SEM). The statistical differences between various experimental groups were evaluated with paired Student’s *t* test. All hypothesis tests were two-tailed and a *p* value of ≤0.05 defined statistical significance.

### Data availability

All relevant data supporting the findings of the study are available in this article and its Supplementary Information files, or from the corresponding authors on request.

## Electronic supplementary material

Supplementary Tables 1 and 2, Supplementary Figure 1

## References

[1] Raposo G, Stoorvogel W (2013). Extracellular vesicles: exosomes, microvesicles, and friends. The Journal of cell biology.

[2] Datta A (2017). Manumycin A suppresses exosome biogenesis and secretion via targeted inhibition of Ras/Raf/ERK1/2 signaling and hnRNP H1 in castration-resistant prostate cancer cells. Cancer Lett.

[3] Khan FM (2018). Inhibition of exosome release by ketotifen enhances sensitivity of cancer cells to doxorubicin. Cancer Biol Ther.

[4] Bianco F (2009). Acid sphingomyelinase activity triggers microparticle release from glial cells. The EMBO journal.

[5] Mire-Sluis AR, Thorpe R (1998). Laboratory protocols for the quantitation of cytokines by bioassay using cytokine responsive cell lines. Journal of immunological methods.

[6] Colombo M, Raposo G, Thery C (2014). Biogenesis, secretion, and intercellular interactions of exosomes and other extracellular vesicles. Annual review of cell and developmental biology.

[7] Delorme-Axford E (2013). Human placental trophoblasts confer viral resistance to recipient cells. Proceedings of the National Academy of Sciences of the United States of America.

[8] Knickelbein JE (2016). Modulation of Immune Responses by Extracellular Vesicles From Retinal Pigment Epithelium. Investigative ophthalmology & visual science.

[9] Sampey GC (2016). Exosomes from HIV-1-infected Cells Stimulate Production of Pro-inflammatory Cytokines through Trans-activating Response (TAR) RNA. The Journal of biological chemistry.

[10] Zhang HG (2006). A membrane form of TNF-alpha presented by exosomes delays T cell activation-induced cell death. Journal of immunology (Baltimore, Md. : 1950).

[11] Chen T, Guo J, Yang M, Zhu X, Cao X (2011). Chemokine-containing exosomes are released from heat-stressed tumor cells via lipid raft-dependent pathway and act as efficient tumor vaccine. Journal of immunology (Baltimore, Md. : 1950).

[12] Zhang B (2016). Exosomes from Human Umbilical Cord Mesenchymal Stem Cells: Identification, Purification, and Biological Characteristics. Stem cells international.

[13] Nickel W, Rabouille C (2009). Mechanisms of regulated unconventional protein secretion. Nature reviews. Molecular cell biology.

[14] Konadu KA (2015). Association of Cytokines With Exosomes in the Plasma of HIV-1-Seropositive Individuals. The Journal of infectious diseases.

[15] Robbins PD, Morelli AE (2014). Regulation of immune responses by extracellular vesicles. Nat Rev Immunol.

[16] Yuana Y, Sturk A, Nieuwland R (2013). Extracellular vesicles in physiological and pathological conditions. Blood Rev.

[17] Saunderson SC, Dunn AC, Crocker PR, McLellan AD (2014). CD169 mediates the capture of exosomes in spleen and lymph node. Blood.

[18] Hao S (2007). Mature dendritic cells pulsed with exosomes stimulate efficient cytotoxic T-lymphocyte responses and antitumour immunity. Immunology.

[19] Dasgupta SK (2009). Lactadherin and clearance of platelet-derived microvesicles. Blood.

[20] Simons M, Raposo G (2009). Exosomes–vesicular carriers for intercellular communication. Current opinion in cell biology.

[21] Calzolari A (2006). TfR2 localizes in lipid raft domains and is released in exosomes to activate signal transduction along the MAPK pathway. Journal of cell science.

[22] Perez-Hernandez D (2013). The intracellular interactome of tetraspanin-enriched microdomains reveals their function as sorting machineries toward exosomes. The Journal of biological chemistry.

[23] Arakelyan A, Fitzgerald W, Zicari S, Vanpouille C, Margolis L (2017). Extracellular Vesicles Carry HIV Env and Facilitate Hiv Infection of Human Lymphoid Tissue. Scientific reports.

[24] Broughton SE, Hercus TR, Lopez AF, Parker MW (2012). Cytokine receptor activation at the cell surface. Current opinion in structural biology.

[25] Eppstein DA, Marsh YV, van der Pas M, Felgner PL, Schreiber AB (1985). Biological activity of liposome-encapsulated murine interferon gamma is mediated by a cell membrane receptor. Proceedings of the National Academy of Sciences of the United States of America.

[26] Margolis LB (1984). Lipid-cell interactions. Liposome adsorption and cell-to-liposome lipid transfer are mediated by the same cell-surface sites. Biochimica et biophysica acta.

[27] Yang ST, Zaitseva E, Chernomordik LV, Melikov K (2010). Cell-penetrating peptide induces leaky fusion of liposomes containing late endosome-specific anionic lipid. Biophysical journal.

[28] Neves JS, Radke AL, Weller PF (2010). Cysteinyl leukotrienes acting via granule membrane-expressed receptors elicit secretion from within cell-free human eosinophil granules. The Journal of allergy and clinical immunology.

[29] Kalra H (2013). Comparative proteomics evaluation of plasma exosome isolation techniques and assessment of the stability of exosomes in normal human blood plasma. Proteomics.

[30] Kumeda N (2017). Characterization of Membrane Integrity and Morphological Stability of Human Salivary Exosomes. Biological & pharmaceutical bulletin.

[31] Grivel JC, Margolis L (2009). Use of human tissue explants to study human infectious agents. Nature protocols.

[32] Lasser, C., Eldh, M. & Lotvall, J. Isolation and characterization of RNA-containing exosomes. *Journal of visualized experiments: JoVE*, e3037 (2012). 10.3791/3037 PMC3369768 22257828

[33] Biancotto A (2007). Abnormal activation and cytokine spectra in lymph nodes of people chronically infected with HIV-1. Blood.

[34] Lisco A (2012). HIV-1 imposes rigidity on blood and semen cytokine networks. American journal of reproductive immunology (New York, N.Y.: 1989).

