# Author Correction: A System of Cytokines Encapsulated in ExtraCellular Vesicles

**DOI:** 10.1038/s41598-020-75735-w

**Published:** 2020-10-29

**Authors:** Wendy Fitzgerald, Michael L. Freeman, Michael M. Lederman, Elena Vasilieva, Roberto Romero, Leonid Margolis

**Affiliations:** 1 Section of Intercellular Interactions, Eunice Kennedy-Shriver National Institute of Child Health and Human Development, National Institutes of Health, Bethesda, USA; 2 Case Western Reserve University, Cleveland, USA; 3 Evdokimov Moscow University of Medicine and Dentistry, Moscow, Russia; 4 Neonatology Branch, Eunice Kennedy-Shriver National Institute of Child Health and Human Development, National Institutes of Health, Detroit, USA

Correction to: *Scientific Reports* 10.1038/s41598-018-27190-x, published online 12 June 2018

In this Article, the legend of Figure 2 is incorrect:

“Distribution of cytokines between the surface and the inner volume of EVs. Fractions of total EV-associated cytokines ± SEM. Samples collected as in Fig. 1. (**a**) Placental villous explants n = 10; (**b**) Amnion explants, n = 10; (**c**) Tonsillar explants n = 5; (**d**) Cervical explants n = 6; (**e**) PPP from healthy donors n = 52; (**f**) Amniotic fluid from 3 donors (**g**) T cells n = 6; (**h**) Monocytes n = 6. *Indicates significant difference p < 0.05 between surface and encapsulated cytokines.”

should read:

“Distribution of cytokines between the surface and the inner volume of EVs. Fractions of total EV-associated cytokines ± SEM. Samples collected as in Fig. 1. (**a**) Placental villous explants n = 10; (**b**) Amnion explants, n = 10; (**c**) Tonsillar explants n = 5; (**d**) Cervical explants n = 6; (**e**) PPP from healthy donors n = 52; (**f**) T cells n = 6; (**g**) Monocytes n = 6; (**h**) Amniotic fluid from 3 donors. *Indicates significant difference p < 0.05 between surface and encapsulated cytokines.”

Consequently, in the Results section under the subheading ‘Cytokines associated with EVs: surface-bound vs. encapsulated’, the referrals to Figure 2 are incorrect.

“In contrast to plasma, in amniotic fluid out of all EV-associated cytokines only two, IL-17 and IFN-γ, were preferentially on the surface of EVs and only small fractions of another three, IL-1α, IL-22, and ITAC, were present on the surface of EVs; whereas 27 other cytokines were almost exclusively inside EVs (Fig. 2f).

For T cells (Fig. 2g) and monocytes (Fig. 2h) fewer cytokines were on the surface of EVs, whereas for both types of cells EV-associated cytokines were predominantly inside the vesicles (25 and 20 respectively).”

should read:

“In contrast to plasma, in amniotic fluid out of all EV-associated cytokines only two, IL-17 and IFN-γ, were preferentially on the surface of EVs and only small fractions of another three, IL-1α, IL-22, and ITAC, were present on the surface of EVs; whereas 27 other cytokines were almost exclusively inside EVs (Fig. 2h).

For T cells (Fig. 2f) and monocytes (Fig. 2g) fewer cytokines were on the surface of EVs, whereas for both types of cells EV-associated cytokines were predominantly inside the vesicles (25 and 20 respectively).”

