# Supplementary material for: A System of Cytokines Encapsulated in ExtraCellular Vesicles
Source: Sci Rep. 2018 Jun 12;8:8973. doi: 10.1038/s41598-018-27190-x (PMC5997670; doi:10.1038/s41598-018-27190-x)
Supplement: Supplementary file 1 — Supplementary Tables 1 and 2, Supplementary Figure 1 [file 41598_2018_27190_MOESM1_ESM.docx]

Supplementary Materials for

**A SYSTEM OF CYTOKINES**

**ENCAPSULATED IN EXTRA-CELLULAR VESICLES**

Wendy Fitzgerald^1^, Michael L. Freeman^2^, Michael M. Lederman^2^, Elena Vasilieva^3^, Roberto Romero^4^* and Leonid Margolis^1^*

^1^Section of Intercellular Interactions, Eunice Kennedy-Shriver National Institute of Child Health and Human Development, National Institutes of Health; ^2^Case-Western University, Cleveland; ^3^Evdokimov Moscow University of Medicine and Dentistry, Moscow; ^4^Neonatology Branch, Eunice Kennedy-Shriver National Institute of Child Health and Human Development, National Institutes of Health

correspondence to: [margolis@helix.nih.gov](mailto:margolis@helix.nih.gov), romeror@mail.nih.gov

**This file includes:**

Supplementary Tables 1 and 2, Supplementary Figure 1

| Supplementary Table 1 Nanosight determination of vesicle size and concentration | | | | |
| --- | --- | --- | --- | --- |
|  | **Concentration (vesicles/ml)** | **Mean (nm)** | **Mode (nm)** | **D90 (nm)** |
| **Plasma** | 3.4 ± 0.7 x 10^11^ | 127 ± 3.2 | 115 ± 1.7 | 187 ± 4.1 |
| **Amniotic Fluid** | 9.9 ± 5.6 x 10^8^ | 149 ± 9.4 | 111 ± 2.8 | 225 ± 40.8 |
| **Placental Villi** | 8.8 ± 0.7 x 10^9^ | 221 ± 6.6 | 152 ± 7.9 | 350 ± 12.1 |
| **Amnion** | 1.9 ± 0.3 x 10^9^ | 151 ± 6.9 | 111 ± 3.8 | 222 ± 16.9 |
| **Tonsil** | 1.2 ± 0.0 x 10^9^ | 190 ± 6.5 | 156 ± 12.4 | 291 ± 47.3 |
| **Cervix** | 3.0 ± 0.5 x 10^9^ | 143 ± 7.5 | 76 ± 19.1 | 238 ± 12.5 |
| **T cells** | 1.6 ± 0.3 x 10^10^ | 127 ± 0.6 | 83 ± 16.7 | 208 ± 32.8 |
| **Monocytes** | 1.1 ± 0.1 x 10^10^ | 123 ± 4.4 | 87 ± 2.7 | 166 ± 6.4 |

Culture supernatants and body fluids were analyzed by NanoSight NS300. Data from the three representative samples was combined to provide averaged values of concentration ± SEM of EV/ml, mean ± SEM particle size (nm), mode ± SEM of particle size (nm), and D90 ± SEM (nm), the size above which 90% of particles are contained. Tissue explant EVs were measured on day 3 of culture for tonsils and cervix (accumulation of EVs over days 1-3), and at day 4 for placental villous and amnion explants (accumulation over days 2-4). T cells and monocyte EVs were measured at day 1 (accumulation over 24 hours).

| Supplementary Table 2 Cytokines in free and EV-associated form in eight different biological systems | | | | | | | | | | | | | | | | |
| --- | --- | --- | --- | --- | --- | --- | --- | --- | --- | --- | --- | --- | --- | --- | --- | --- |
|  | **Plasma free** | **Plasma EV** | **Amniotic Fluid free** | **Amniotic Fluid EV** | **Villi free** | **Villi EV** | **Amnion free** | **Amnion EV** | **Tonsil free** | **Tonsil EV** | **Cervix free** | **Cervix EV** | **T Cells free** | **T Cells EV** | **Monocytes free** | **Monocytes EV** |
| IL-1α | **0.5**  **±0.2** | **2.8**  **±0.4** | **13.9** | **2.5** | **18.8**  **±3.5** | **9.4**  **±1.8** | **3.4**  **±2.0** | **5.5**  **±1.4** | **964.2**  **±170.9** | **64.8**  **±13.9** | **67.2**  **±15.8** | **15.1**  **±4.2** | **--** | **1.3**  **±0.6** | **0.1**  **±0.2** | **1.3**  **±0.8** |
| IL-1β | **7.5**  **±1.3** | **5.5**  **±1.6** | **4.3** | **7.0** | **56.0**  **±7.9** | **21.0**  **±2.8** | **22.1**  **±4.3** | **15.4**  **±3.5** | **166.8**  **±42.8** | **23.9**  **±4.1** | **15.8**  **±3.0** | **17.2**  **±3.0** | **0.1**  **±0.1** | **2.7**  **±1.1** | **4.4**  **±2.9** | **3.9**  **±2.1** |
| IL-2 | **0.1**  **±0.1** | **0.4**  **±0.2** | **--** | **--** | **19.7**  **±4.7** | **18.3**  **±5.1** | **3.0**  **±2.8** | **9.1**  **±3.0** | **26.7**  **±3.1** | **103.2**  **±27.0** | **1.4**  **±0.6** | **10.7**  **±1.8** | **--** | **0.4**  **±0.4** | **--** | **--** |
| IL-4 | **--** | **--** | **--** | **13.3** | **--** | **--** | **--** | **--** | **44.8**  **±5.5** | **228.0**  **±44.3** | **--** | **50.8**  **±23.5** | **--** | **15.4**  **±6.9** | **--** | **11.1**  **±7.6** |
| IL-6 | **1.9**  **±1.8** | **0.3**  **±0.1** | **468.2** | **12.3** | **277,539.4±42,585.7** | **2,200.1**  **±441.6** | **43,723.8**  **±17,050.6** | **339.6**  **±166.7** | **52,364.3**  **±2,892.7** | **1,0229.6**  **±2,913.9** | **754,211.0**  **±106,343.5** | **10,854.8**  **±3,224.9** | **--** | **0.1**  **±0.1** | **8.4**  **±2.2** | **0.1**  **±0.0** |
| IL-7 | **--** | **--** | **3.3** | **2.3** | **--** | **--** | **--** | **--** | **18.2**  **±5.3** | **47.1**  **±12.4** | **7.6**  **±0.7** | **5.5**  **±1.2** | **--** | **0.9**  **±0.6** | **--** | **1.0**  **±0.7** |
| IL-8 | **1.1**  **±0.3** | **0.2**  **±0.1** | **660.4** | **8.4** | **9,1826.1**  **±5,396.8** | **2,020.3**  **±254.0** | **46,619.1±9,834.0** | **728.1**  **±286.6** | **14,278.5**  **±2,346.9** | **31,466.4**  **±5,635.1** | **203,226.8**  **±34,124.3** | **5,934.0**  **±1,515.7** | **6.7**  **±2.7** | **1.4**  **±0.8** | **389.0**  **±247.1** | **66.0**  **±44.9** |
| IL-10 | **--** | **12.9**  **±5.0** | **--** | **2.9** | **55.5**  **±6.9** | **22.5**  **±1.8** | **74.4**  **±33.6** | **13.2**  **±2.1** | **57.6**  **±11.1** | **1,349.3**  **±159.1** | **42.2**  **±22.9** | **33.9**  **±22.4** | **--** | **43.3**  **±17.1** | **--** | **45.1**  **±26.1** |
| IL-12 p70 | **3.0**  **±2.0** | **23.8**  **±14.3** | **--** | **1.3** | **--** | **--** | **--** | **--** | **49.7**  **±6.8** | **152.5**  **±30.8** | **--** | **4.7**  **±1.1** | **--** | **--** | **--** | **--** |
| IL-13 | **52.5**  **±20.8** | **71.9**  **±25.0** | **--** | **--** | **433.3**  **±66.5** | **166.5**  **±31.4** | **176.8**  **±52.7** | **83.4**  **±29.0** | **1,049.8**  **±76.9** | **480.0**  **±114.7** | **77.1**  **±29.2** | **11.4**  **±6.4** | **--** | **--** | **--** | **--** |
| IL-15 | **5.7**  **±1.4** | **6.6**  **±1.1** | **75.4** | **1.0** | **83.9**  **±11.4** | **7.1**  **±1.2** | **6.5**  **±5.0** | **3.0**  **±0.7** | **3.6**  **±2.0** | **--** | **27.3**  **±6.6** | **5.7**  **±2.5** | **--** | **20.8**  **±20.6** | **--** | **0.1**  **±0.0** |
| IL-16 | **105.7**  **±10.2** | **21.4**  **±2.4** | **229.4** | **62.3** | **1,417.3**  **±222.4** | **373.1**  **±61.1** | **145.5**  **±123.0** | **172.8**  **±46.3** | **10,547.8**  **±2,529.3** | **1,766.8**  **±630.2** | **1,742.4**  **±518.0** | **201.8**  **±171.6** | **50.4**  **±23.6** | **62.9**  **±24.7** | **91.9**  **±68.4** | **51.9**  **±27.8** |
| IL-17 | **--** | **--** | **--** | **5.9** | **--** | **--** | **--** | **--** | **555.5**  **±268.6** | **569.6**  **±298.6** | **3.9**  **±1.8** | **6.3**  **±2.1** | **--** | **13.4**  **±6.1** | **--** | **14.3**  **±9.4** |
| IL-18 | **306.6**  **±20.7** | **14.1**  **±1.3** | **1.9** | **0.5** | **21.6±2.6** | **5.7**  **±0.6** | **2.5**  **±1.5** | **2.8**  **±0.5** | **286.8**  **±100.2** | **29.8**  **±5.9** | **10.2**  **±5.9** | **1.1**  **±0.2** | **--** | **1.5**  **±0.7** | **--** | **0.7**  **±0.4** |
| IL-21 | **254.0**  **±93.0** | **992.0**  **±493.5** | **8,093.9** | **3,040.1** | **--** | **--** | **--** | **--** | **9,690.4**  **±213.8** | **22,653.6**  **±3,247.4** | **105.6**  **±62.6** | **188.6**  **±70.3** | **6.7**  **±6.7** | **686.9**  **±123.9** | **0.2**  **±0.2** | **645.5**  **±242.0** |
| IL-22 | **36.2**  **±11.2** | **205.9**  **±30.2** | **84.3** | **256.9** | **--** | **--** | **--** | **--** | **746.1**  **±134.9** | **2,530.0**  **±320.0** | **53.9**  **±53.9** | **547.8**  **±165.2** | **--** | **110.4**  **±34.5** | **--** | **86.6**  **±54.5** |
| IL-33 | **4.2**  **±3.0** | **284.9**  **±45.6** | **523** | **237.6** | **1,082.5**  **±240.0** | **1,128.0**  **±217.6** | **606.0**  **±217.0** | **759.4**  **±182.4** | **1,048.2**  **±208.1** | **17,110.9**  **±5,317.3** | **88.0**  **±41.0** | **435.6**  **±186.8** | **35.7**  **±22.6** | **48.0**  **±33.2** | **60.3**  **±58.4** | **78.1**  **±80.1** |
| Calgran A | **50.3**  **±25.7** | **216.7**  **±109.5** | **94.3** | **30.3** | **1,109.9**  **±193.3** | **353.6**  **±88.6** | **172.8**  **±96.5** | **201.5**  **±43.6** | **613.5**  **±156.5** | **298.2**  **±87.8** | **704.5**  **±294.9** | **162.0**  **±108.2** | **--** | **--** | **--** | **--** |
| Eotaxin | **17,833.3**  **±2,336.5** | **5,704.2**  **±889.1** | **1,826.9** | **3124.0** | **129.6**  **±26.6** | **103.9**  **±42.4** | **--** | **57.7**  **±38.5** | **14,193.5**  **±872.3** | **9,972.0**  **±2,160.0** | **24.7**  **±9.0** | **78.9**  **±18.1** | **--** | **112.9**  **±56.9** | **--** | **126.4**  **±83.3** |
| GM-CSF | **0.1**  **±0.1** | **0.1**  **±0.1** | **6.9** | **--** | **432.4**  **±177.4** | **15.0**  **±2.5** | **6.5**  **±3.0** | **3.2**  **±1.6** | **9,733.1**  **±2,544.3** | **221.5**  **±45.8** | **406.0**  **±98.3** | **8.4**  **±5.3** | **0.1**  **±0.1** | **3.1**  **±1.2** | **--** | **3.1**  **±2.2** |
| Gro-α | **1.7**  **±0.9** | **15.4**  **±8.3** | **938.5** | **151.2** | **30,532.5**  **±5,048.7** | **814.1**  **±172.0** | **34,941.6**  **±8,388.9** | **1,096.2**  **±284.8** | **9,980.9**  **±804.2** | **19,500.0**  **±4,216.3** | **74,816.7**  **±14,851.5** | **18,722.6**  **±10,385.5** | **--** | **33.9**  **±17.0** | **--** | **44.2**  **±28.1** |
| IFN-γ | **25.1**  **±16.9** | **81.5**  **±41.6** | **190.7** | **182.8** | **43.3**  **±19.4** | **23.0**  **±10.0** | **--** | **5.7**  **±3.7** | **757.6**  **±90.9** | **2,763.4**  **±422.0** | **69.5**  **±18.4** | **416.8**  **±138.9** | **0.9**  **±0.7** | **854.9**  **±383.0** | **4.3**  **±3.8** | **592.1**  **±409.8** |
| IP-10 | **3,139.2**  **±452.8** | **774.6**  **±128.0** | **82,517.2** | **29,762.4** | **138,785.1±35,770.6** | **8,585.3**  **±2,089.8** | **7,397.5**  **±3,895.3** | **351.1**  **±163.7** | **75,520.5**  **±46,110.9** | **33,550.6**  **±26,029.6** | **31,569.9**  **±21,364.2** | **2,814.9**  **±1,739.0** | **--** | **10.0**  **±4.6** | **46.8**  **±42.0** | **52.3**  **±36.3** |
| I-TAC | **--** | **9.6**  **±5.6** | **403.7** | **340.2** | **2,067.7**  **±290.6** | **490.2**  **±72.7** | **120.9**  **±87.5** | **87.5**  **±37.8** | **581.7**  **±79.1** | **1,286.2**  **±211.1** | **160.8**  **±133.7** | **26.0**  **±7.8** | **--** | **23.6**  **±15.1** | **--** | **20.8**  **±20.2** |
| M-CSF | **1.2**  **±0.4** | **11.1**  **±1.6** | **2.7** | **2.7** | **183.6**  **±23.9** | **60.7**  **±5.4** | **19.2**  **±7.1** | **35.6**  **±4.5** | **248.5**  **±30.3** | **257.4**  **±85.6** | **118.4**  **±21.2** | **16.8**  **±6.0** | **--** | **10.6**  **±4.8** | **--** | **13.0**  **±8.2** |
| MCP-1 | **328.5**  **±31.3** | **95.0**  **±6.3** | **1,143.0** | **76.1** | **27,898.8**  **±7,614.0** | **500.7**  **±107.4** | **3,129.1**  **±1,425.6** | **23.9**  **±11.9** | **36,849.5**  **±2,603.6** | **16,815.3**  **±3,584.5** | **36,858.1**  **±8,855.0** | **754.8**  **±161.3** | **--** | **--** | **5.3**  **±4.2** | **--** |
| MIG | **991.1**  **±208.6** | **113.0**  **±35.0** | **361.0** | **44.9** | **1,764.4**  **±748.8** | **378.5**  **±58.4** | **260.8**  **±258.2** | **39.4**  **±23.4** | **10,446.6**  **±6,299.3** | **2,223.5**  **±949.3** | **16,598.1**  **±12,401.3** | **648.6**  **±209.3** | **--** | **139.7**  **±62.7** | **--** | **99.8**  **±66.2** |
| MIP-1α | **18.1**  **±4.0** | **43.5**  **±5.1** | **7.6** | **--** | **1,112.1**  **±263.4** | **175.8**  **±23.1** | **1,546.2**  **±552.6** | **118.8**  **±20.6** | **575.4**  **±65.3** | **232.8**  **±60.6** | **3,758.8**  **±2,409.8** | **263.7**  **±160.2** | **0.4**  **±0.3** | **10.4**  **±4.5** | **41.0**  **±24.6** | **13.9**  **±9.0** |
| MIP-1β | **66.0**  **±3.8** | **27.6**  **±1.4** | **29.0** | **2.3** | **1,993.3**  **±336.4** | **78.3**  **±11.3** | **1,810.3**  **±596.5** | **59.4**  **±19.2** | **666.5**  **±67.4** | **229.8**  **±78.4** | **716.5**  **±226.2** | **62.1**  **±27.6** | **1.4**  **±1.0** | **16.8**  **±14.0** | **520.1**  **±306.4** | **178.7**  **±128.0** |
| MIP-3α | **--** | **--** | **--** | **382.6** | **6,133.4**  **±1,660.7** | **92.1**  **±33.1** | **233.5**  **±177.7** | **--** | **6,122.2**  **±1,670.8** | **4,960.3**  **±702.0** | **3,691.0**  **±1,723.4** | **28.1**  **±28.1** | **--** | **--** | **--** | **--** |
| RANTES | **4,408.3**  **±615.3** | **3,599.6**  **±435.8** | **14.4** | **11.0** | **412.4**  **±105.1** | **6.5**  **±4.2** | **5.5**  **±3.8** | **--** | **1,049.2**  **±175.9** | **2,162.8**  **±1,400.3** | **363.6**  **±99.9** | **54.9**  **±36.3** | **--** | **--** | **388.8**  **±263.5** | **358.5**  **±244.0** |
| TGF-β | **6.4**  **±4.9** | **36.1**  **±22.5** | **9.0** | **19.5** | **16.2**  **±5.2** | **7.4**  **±3.3** | **3.1**  **±3.1** | **1.5**  **±1.2** | **184.1**  **±10.5** | **189.4**  **±12.6** | **165.7**  **±50.4** | **36.6**  **±28.3** | **--** | **7.3**  **±4.0** | **--** | **2.5**  **±2.2** |
| TNF-α | **4.9**  **±0.8** | **6.5**  **±1.5** | **0.9** | **0.5** | **24.4**  **±4.1** | **21.4**  **±4.3** | **9.0**  **±4.0** | **10.9**  **±3.1** | **83.5**  **±8.2** | **204.6**  **±14.5** | **4.0**  **±1.2** | **13.5**  **±5.5** | **0.1**  **±0.1** | **1.6**  **±0.6** | **1.8**  **±1.3** | **1.6**  **±0.8** |

Mean concentration ± SEM (pg/ml) of 33 cytokines was measured by in-house multiplexed bead-based assay. Culture supernatants or body fluids were treated with ExoQuick to sediment EVs. Measurements were made on supernatants free of EV and on lysed EV fractions. Note that after EV isolation, EV pellets are resuspended in the original volume of media. Concentration of cytokines inside EVs, whose total volume is small, is orders of magnitude higher than indicated in the table. Means are based on n=52 for PPP, n=3 for amniotic fluid, n=10 for placental villi and amnion explants, n=5 for tonsillar explants, n=6 for cervical explants, and n=6 for T cells and monocytes.

**a**

**b**

**Supplementary Figure 1.** (**a)** Cytokines in free form and EV-associated are expressed as percent of total cytokine released ± SEM for tonsil explants at day 3, 6, and 9 of culture, n=5. Blue bars: cytokine in “free” form, red: EV-associated. (**b)** Fractions of total EV-associated cytokines ± SEM, n=5. Blue bars: cytokine on EV surface, red: cytokine EV-encapsulated. Note that the cytokines maintain similar distributions over time.
